# Nonlinear Resonance Vibration Assessment to Evaluate the Freezing and Thawing Resistance of Concrete

**DOI:** 10.3390/ma12020325

**Published:** 2019-01-21

**Authors:** Jae Hong Kim, Sun-Jong Park, Hong Jae Yim

**Affiliations:** 1 Department of Civil and Environmental Engineering, Korean Advanced Institute for Science and Technology, 291 Daehak-ro, Yuseong-gu, Daejeon 34141, Korea; Jaekim@gmail.com; 2 Korea Institute of Nuclear Safety, 62 Gwahak-ro, Yuseong-gu, Daejeon 34142, Korea; sunjongpark@kins.re.kr; 3 Department of Construction and Disaster Prevention Engineering, Kyungpook National University, Sangju-si 37224, Korea

**Keywords:** concrete, freezing and thawing, nonlinear ultrasound, durability

## Abstract

Under cold environments, the freezing and thawing cycles of water in concrete reduce the lifetime and durability of concrete structures. For enhanced freezing and thawing resistance, entrained air voids are generally required, but malfunctioning of air entrainment is sometimes reported in the field. To evaluate the quality of air entrainment, this study proposes a nondestructive method that is a preceding evaluation before damage to the concrete. A nonlinear resonance vibration method is adopted in samples having an identical air void content. The durable concrete sample with resistance to freezing and thawing cycles shows higher nonlinearity in its resonance. Thus, the quality of air entrainment and, furthermore, the potential freezing and thawing resistance can possibly be evaluated by measuring the nonlinearity parameter of the concrete, which is preliminary study to attempt the preceding evaluation of freezing and thawing resistance using nondestructive method.

## 1. Introduction

The freezing of concrete causes the volume expansion of the water trapped in the capillary pores. Powers’ hypothesis indicates that ice formation in pores ranging from 0.01 to 10 μm is associated with hydraulic pressure from the constraints of space [1]. Scherer and Valenza argued that the crystallization pressure of ice also induces internal stress during freezing [2]. The pressure consequently causes cracks with multiple cycles of freezing and thawing, or it allows adjacent water to be expelled into entrained air voids [3,4,5]. 

Air entrainment is required to provide concrete with freezing and thawing resistance. Air contents of 4.5% for normal concrete or 3.5% for high-strength concrete (over 35 MPa) are generally asked for in the field [6]. Nevertheless, failures in freezing and thawing resistance in lab testing and field performance are sometimes reported, and freezing and thawing resistance of various types of concrete was evaluated to investigate the effect of included cementitious materials and recycled aggregates based on the experimental results [7,8,9,10]. The quality of air entrainment needs more sophisticated evaluation using a spacing factor of the entrained air voids. ASTM C 457/C 457M-16 evaluates the spacing factor, L, as half of the longest distance between two entrained air voids given that they are monosized and equally distributed [11]. The spacing information is obtained by petrography of polished sections of concrete. Powers’ test results supported air entrainment with L of <250 μm as effective for conferring freezing and thawing resistance [1]. Finely distributed entrained air voids ranging from 50 µm to 200 µm are beneficial for freezing and thawing durability [12]. Other factors to describe the spacing information of entrained air voids have been also proposed [13,14,15,16,17,18]. However, the spacing factors may be ill-defined with outliers, and the preparation of sectional petrography is also prone to error. This study proposes an alternative method to evaluate the quality of air entrainment. The use of nonlinear ultrasound allows us to nondestructively evaluate the entrained air void system. 

Nondestructive assessment using an acoustic method was reported and the evaluation of void and crack due to freezing and thawing cycle was performed by X-ray computed tomography [19]. Nonlinear ultrasound shows better sensitivity for probing local contact-type defects in composites, including cement-based materials [20,21,22,23,24]. The opening and closing of the defects or voids is involved with stress at a microscale. The application of the nonlinear ultrasonic method in cement-based materials has also been reported for the evaluation of the strength of concrete [25], damage due to alkali–silica reaction [26], the degree of thermal damage in concrete [20,21,27,28,29], carbonation assessment [30], and the accumulated damage due to freezing and thawing cycles [31,32].

The above applications of nonlinear ultrasound evaluate the progression of damage related to the strength and durability of concrete, but the prediction or preceding evaluation of a sample’s performance has not been attempted. This study attempts to evaluate the resistance of freezing and thawing damage of concrete before occurrence of damage in sound concrete, and the verification of the proposed method was performed for the first time. Nonlinear resonance vibration is herein adopted to assess the potential freezing and thawing resistance and the quality of air entrainment in concrete. Finely distributed air voids, which contribute to better freezing and thawing resistance, are one of the contact-type defects affecting the characteristics of the nonlinear ultrasound. As a preliminary study on preceding evaluation using a nondestructive method, the sensitivity and efficiency of the proposed method are discussed based on experimental results from four concrete samples.

## 2. Nonlinear Resonance Vibration 

**Figure 1:**
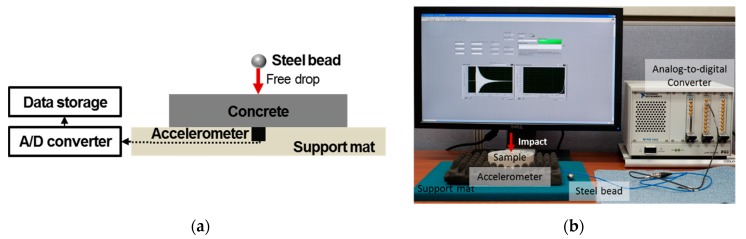

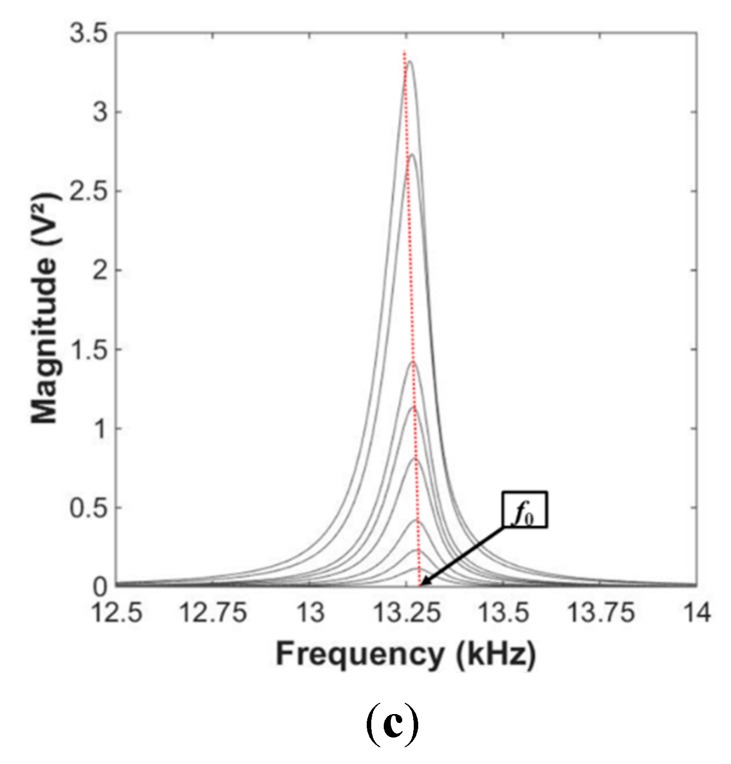
(**a**) Schematic diagram of the nonlinear resonance vibration method; (**b**) experimental setup; (**c**) amplitude-dependent resonant frequency shift for specimen *A*1.

Figure 1 shows a schematic diagram and the experimental setup used to measure the nonlinear resonance vibration of a specimen. The nonlinear resonance vibration method, which is a type of nonlinear ultrasonic technique, was applied for the preceding evaluation of freezing and thawing damage occurring in the concrete. A concrete disk specimen with 25 mm in thickness and 100 mm in diameter was placed on a soft mat, where free vibration of the specimen was allowed by dropping a steel bead weighing 13.8 g from different heights above the specimen at the marked center of the disk. The impact-induced vibration was measured using an accelerometer (PCB353B15; PCB Piezotronics Inc., Depew, NY, USA). The signal was then converted via an analogue-to-digital converter (NI PXI 4472-B; National Instruments Corp., Austin, TX, USA) with a 100 kS/s sampling rate for 50 ms. A fast Fourier transform algorithm converted the waveform signal into the frequency domain, permitting the determination of the resonant frequency of the disk specimen.

Increasing the free-fall height of the steel bead gave a higher amplitude of the impact resonance. The resonant frequency of the disk specimen increased with a higher amplitude of the impact. Figure 1c shows an example of the amplitude-dependent resonant frequency shift. It should be noted that a Hookean solid ideally shows independence of the impact amplitude. A total of 20 impact amplitudes were tested for each specimen, and a shifting of the resonance frequencies of the intact concrete specimen was commonly observed. The degree of the amplitude-dependent resonance frequency shift can be evaluated with a hysteretic nonlinearity parameter. The constitutive equation of a nonlinear elastic solid [33,34] is as follows:

(1) $$M(\varepsilon ,\dot{\varepsilon })=M_{0}(1-\beta \varepsilon -\delta \varepsilon^{2}\cdots )+M_{0}\left\{-\alpha \left[\Delta \varepsilon +\varepsilon (t)\mathrm{sign}(\dot{\varepsilon })\right]+\cdots \right\}$$

where *ε* = d*ε/dt* is the strain rate, Δ*ε* is the strain amplitude change over the previous period, sign(*ε*) = +1 if *ε* > 0 or −1 if *ε* < 0, and *β* and *δ* are the quadratic and cubic orders of nonlinearity, respectively. The hysteretic nonlinearity parameter, expressed by *α*, has been previously used to evaluate amplitude-dependent resonance frequency shifts [27,28,29]. The measured frequency shift then determines the hysteretic nonlinearity parameter as follows:

(2) $$\frac{f_{0}-f}{f_{0}}=\alpha \Delta \varepsilon$$

where *f*_0_ is the linear resonant frequency and *f* is the biased resonance from a higher amplitude of the impact strain (*ε*). The background theory assumes that the resonant frequency is proportional to the amplitude of strain.

## 3. Experiments and Discussions

Table 1 reports the mix proportions of four types of cylindrical concrete samples, where the diameter and height of each sample were 100 mm and 200 mm, respectively. The water-to-binder ratios (*w*/*b*) of samples *A*, *B*, *C*, and *D* were 0.34, 0.41, 0.50, and 0.60, respectively. The compressive strength of each sample was between 29 MPa and 63 MPa after 28 days. The binders for samples *A* and *B* consisted of Type I Portland cement having 3.15 specific gravity, supplementary cementitious materials such as fly ash and ground granulated blast-furnace slag (GGBFS), and calcium sulfoaluminate-type expander (Expander in Table 1), while samples *C* and *D* used only the cement. Table 2 shows the oxide composition of the used materials (cement, fly ash, and GGBFS). The maximum sizes of sand and gravel were 4 mm and 10 mm, respectively. The specific gravities of sand was 2.60 in an oven-dry condition and 2.62 in a surface-dry saturated condition, while that of coarse sand and gravel were 2.72 in an oven dry condition and 2.73 in a surface-dry saturated condition. A high-range water-reducing admixture (HRWRA in Table 1) including an air-entraining admixture was also used to generate entrained air voids in samples *A* and *B*. The HRWRA used was a polycarboxylate-based admixture with a solid content of 30% by mass (ADVA 128). Accordingly, samples *A* and *B* incorporated 3.5% entrained air, which is the amount required for high-strength concrete in field specifications. The amount of air voids in the fresh mix of all samples was measured three times following the ASTM C 231 pressure method [35]. Samples *C* and *D* were mixed without air entrainment, and they were considered the control samples. Represented values of air content in Table 1 are measured ranges by three times tests. For identical entrained air contents in samples *A* and *B*, the mix proportions were selectively determined based on several pretests.

**Figure 2:**
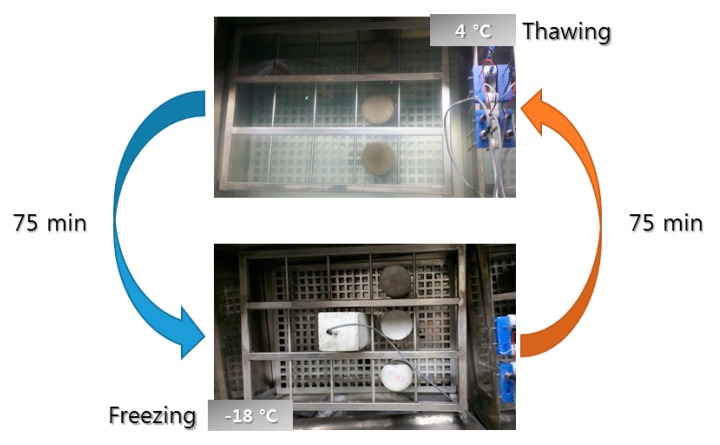
Cycles of freezing and thawing damage of concrete.

Each sample group having different mix proportions was mixed for 20 min and fabricated cylindrical samples were cured under water at 21 °C for 28 days. After curing, samples were moved to water at 4 °C in freezing and thawing equipment as saturated conditions. The freezing and thawing resistance of the samples was tested in accordance with ASTM C 666 [36]. Procedure B was adopted: the samples were thawed in 4 °C water for 75 min and then frozen in air with the temperature set to −18 °C. A cycle lasted for 150 min, and the cycles of freezing and thawing damage are described in Figure 2. 

**Figure 3:**
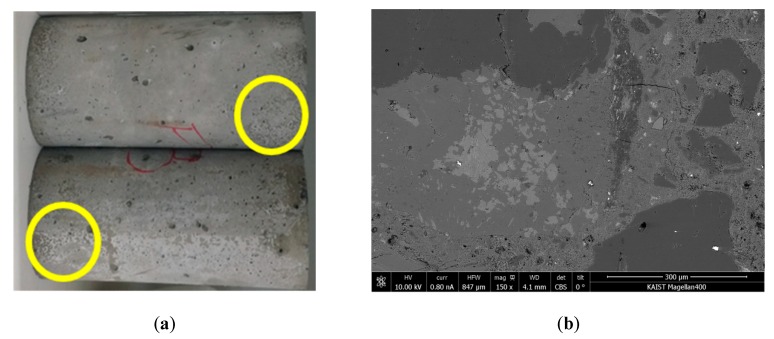

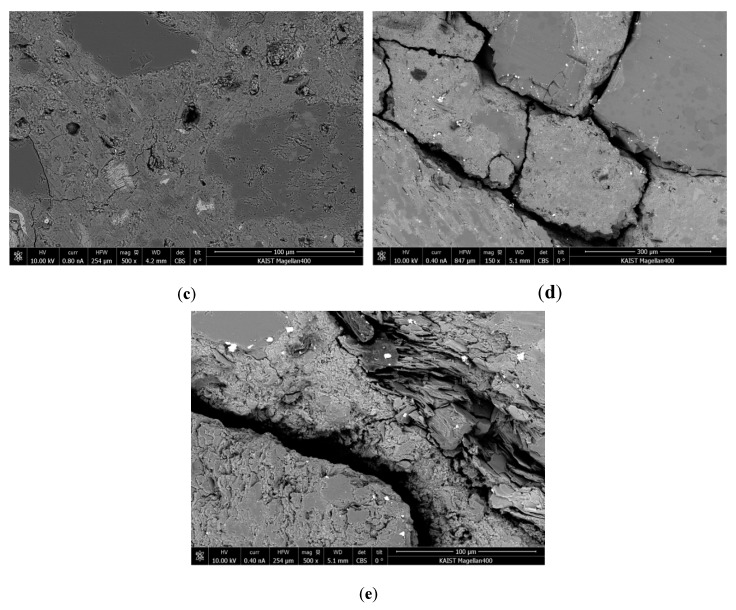
(**a**) Freezing and thawing damage to the concrete surface of sample *A*; SEM micrographs of undamaged concrete before cycles of freezing and thawing with a 150× magnification (**b**) and a 500× magnification (**c**), and damaged concrete after 70 cycles of freezing and thawing with a 150× magnification (**d**) and 500× magnification (**e**).

**Figure 4:**
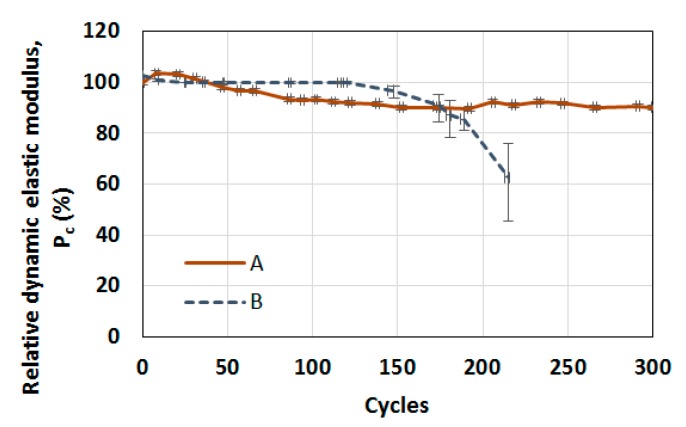
The relative dynamic modulus of elasticity in each cycle for a comparison between samples *A* and *B*.

Damage to the concrete occurred during the accelerated freezing and thawing cycles. Figure 3 shows the appearance of a concrete sample obtained before cycles (sound condition) and after 70 cycles of freezing and thawing. As shown in Figure 3a, honeycombs or multiscale defects were observed on the concrete surface. A piece of the sample was extracted for microscale imaging. The obtained scanning electron microscope (SEM) images at a 150× and a 500× magnifications are shown in Figure 3b,c for undamaged conditions, respectively and Figure 3d,e for damaged conditions, respectively. The processing details for obtaining an SEM image were described in a previous paper by Yim et al. [31]. After 70 cycles, SEM micrographs with the 150× magnification indicated contact-type defects (e.g., microvoids), which mainly occurred in the cement paste that was surrounded by aggregates. The interfacial transition zone, including the defects, was vulnerable to freezing and thawing damage. Several dimensions of crack widths and openings (also called microvoids) were observed at the 500× magnification. These defects were expected to increase in size and number with continued freezing and thawing cycles. While non-air-entrained concrete showed poor resistance, even the durability factor of air-entrained sample *B* was not acceptable, as shown in Figure 4. The durability factor (*DF*) was calculated as *DF* = *P_N_·N*/300, where *P_N_* is the relative dynamic modulus of elasticity at *N* cycles [37]. In summary, only sample *A* contained good-quality entrained air for the freezing and thawing resistance.

**Figure 5:**
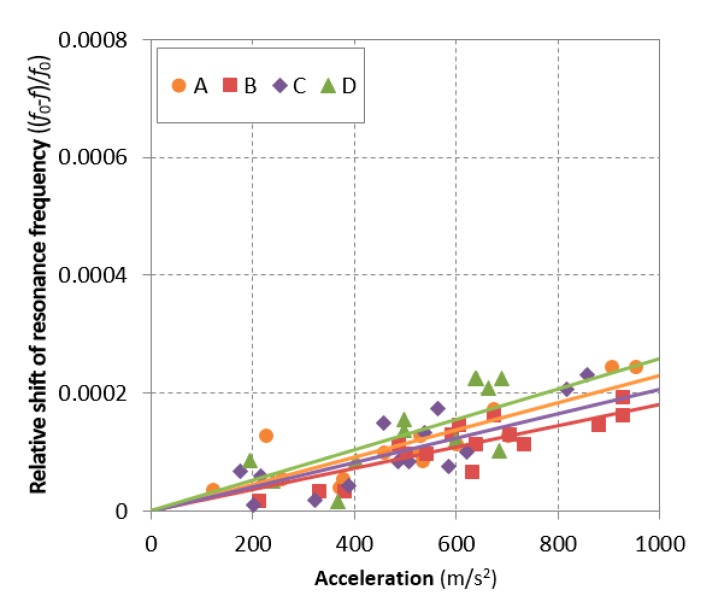
Relative resonant frequency for all samples up to 1 km/s^2^ acceleration.

After curing for 28 days, a total of four disk specimens per sample were fabricated by cutting the cylindrical concrete samples to measure the hysteretic nonlinearity parameter, where the cylindrical concrete was not the damaged sample under freezing and thawing cycles but the additionally prepared sample for nondestructive tests. Each disk specimen was 25 mm thick and 100 mm in diameter. The resonant frequency at the reference state (*f*_0_) was approximately 12 to 14 kHz, where the amplitude of the reference impact was approximately 200 m/s^2^ in acceleration. Figure 5 compares the results of the measured resonance frequencies. The vertical axis represents the relative shift of the measured resonant frequency computed using Equation (2). The measured acceleration of the generated impact is arranged on the horizontal axis. The upper bound of the applied acceleration was 1000 m/s^2^, which was the maximum damage range employed in previous studies to evaluate the progression of damage [27,28,29]. The shift of all samples was small enough to be called sound—less than 0.03% relative shift at an acceleration of 1 km/s^2^, regardless of air entrainment—while a fire-damaged sample in a previous study [27,28,29] showed a 3-fold higher shift. Accordingly, it can be concluded that the nonlinear resonance vibration at the range of 1 km/s^2^ acceleration is not sensitive to the entrained air voids. Such a small amplitude is not sufficient to activate the contact-type defects or voids in concrete. This means that the previously proposed range for damage evaluation of concrete samples using measurement of nonlinearity parameter is a meaningless and uninfluential range to preceding evaluation of the undamaged concrete sample which is zero cycle (100% *P*_c_ in Figure 4), and more energy is required to impact vibration in undamaged concrete before freezing and thawing cycles.

**Figure 6:**
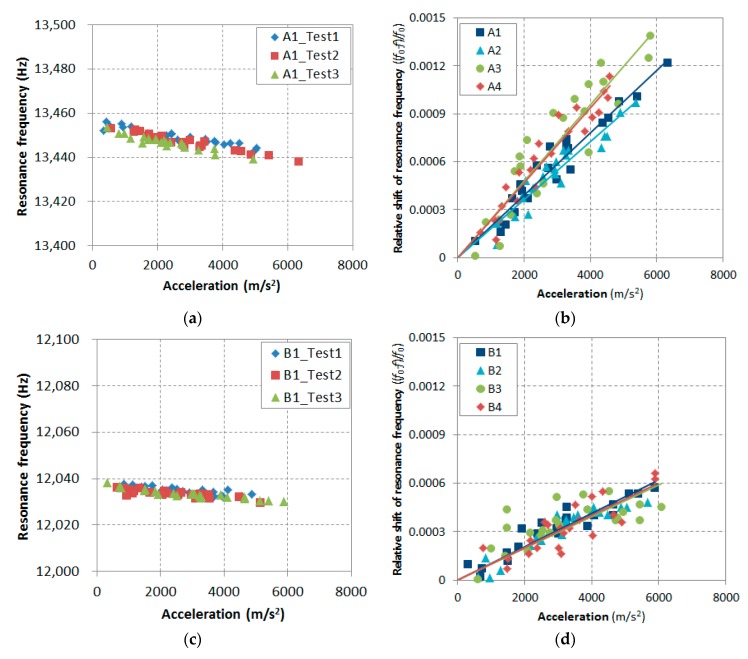
Repeatability and reproducibility test results at a higher acceleration.

To implement the initial evaluation of the undamaged concrete, the amplitude of the impact was increased up to 6 km/s^2^ acceleration to measure the nonlinearity parameter responding to the entrained void. The reproducibility in the high range was firstly examined in Figure 6. A total of three independent measurements on four disk specimens each of sample group *A* (labeled *A*1, *A*2, *A*3, and *A*4) and group *B* (labeled *B*1, *B*2, *B*3, and *B*4) were tested, and the measured resonant frequencies of *A*1 and *B*1 with respect to the applied acceleration were recorded as shown in Figure 6a,c. The linear proportionality of the resonant frequency and acceleration holds for all repeated measurements, where the coefficient of determination was over 0.91. The reproducibility of the measurements was tested with four disk specimens; thus, Figure 6b,d show the variation in the relative resonance frequency shift. The relative shift is a little scattered—compare its variation between the ranges of 1 km/s^2^ and 6 km/s^2^ in Figure 5 and Figure 6. Nevertheless, each measurement shows a coherent shift on the samples *A* and *B*, and they were also consistent on the other samples. 

**Figure 7:**
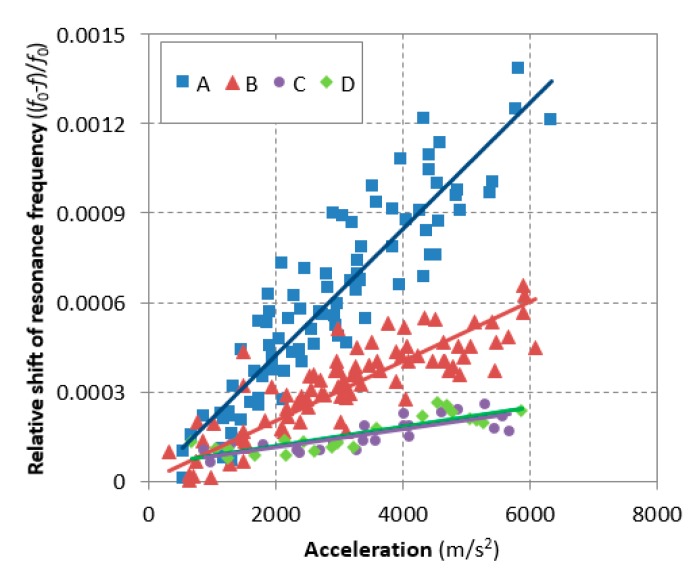
Total result of the relative resonance frequency of sample group *A*, group *B*, group *C*, and group *D* and the linear regression for calculating the hysteretic nonlinearity parameters.

Figure 7 finally compares the results of sample groups *A* and *B* including groups *C* and *D* as the control sample. The relative shifts based on the linear curve fitting to the total measurements were 0.09% and 0.04% for sample groups *A* and *B*, respectively, at 4 km/s^2^ acceleration. The nonlinearity parameters calculated using Equation (2) were 0.242 × 10^−6^ and 0.092 × 10^−6^ for samples *A* and *B*, respectively, where Δ*ε* is proportional to the peak amplitude of the acceleration and then compensated in the calculation. The relative shift of sample *B* was still in the trend of the sound state; this can be seen from the fact that extrapolating the linear curve in Figure 3 meets with the current measurement. The other samples *C* and *D*, having no entrained air, were also in the trend of the sound state. This result shows sensitivity of the nonlinear resonance vibration method on entrained air in undamaged concrete. The nonlinearity parameter of sample *A* was 2.6-times larger than the result of sample *B* and its difference was 163%. This result indicates that Sample *A* has inherent potential contact-type defects that correspond to the entrained void when compared to Sample *B*. The measurement of air content in concrete has been accepted to evaluate the freezing and thawing resistance [38], and the guideline is still valid in various specifications of ordinary concrete. However, recent study reported the importance of spacing and size of air voids in concrete on resistance of freezing and thawing [17,39]. Accordingly, although both samples (*A* and *B*) had the similar amount of air content at the fresh state, the other factor (i.e. inadequate size of the air voids) in Sample *B* caused the hardships to be detected by the nonlinear resonance vibration method, and they also provided deficient freezing and thawing resistance.

## 4. Conclusions

This study proposed a nondestructive method to evaluate the quality of air entrainment in concrete. Finely distributed air voids are important for securing freezing and thawing resistance in concrete. An example mix is reported which indicates that air voids that are inadequate in size are not beneficial, despite having an acceptable air content. The nonlinearity parameter of the example mix, measured via impact resonance vibration, is in the range of sound concrete having no contact-type defects. Non-air-entrained concrete also shows the same range for the nonlinearity parameter. In contrast, good-quality entrained air voids increase the nonlinearity parameter at a high amplitude (up to 6 km/s^2^ acceleration) because they are contact-type defects sensitively responding to the nonlinear ultrasound. Even though experimental result hardly provides a qualitative evaluation, as preliminary study, the proposed method can allow us to evaluate the quality of air entrainment, resulting in a preceding evaluation of the freezing and thawing resistance of concrete.

## Figures and Tables

**Table 1:** Mix proportions of concrete samples.

Label	*A*	*B*	*C*	*D*
Compressive strength [MPa]	63	48	52	29
Air content [%]	3.5 ± 1.5	3.5 ± 1.5	0.2 ± 0.1	0.6 ± 0.2
*w*/*b*	0.34	0.41	0.50	0.60
Water [kg/m^3^]	165	175	160	171
Cement [kg/m^3^]	368	292	320	285
Fly ash [kg/m^3^]	-	43	-	-
GGBFS * [kg/m^3^]	123	86	-	-
Sand [kg/m^3^]	482	493	744	744
Coarse sand [kg/m^3^]	324	488	-	-
Gravel [kg/m^3^]	880	715	1,100	1,100
Expander [kg/m^3^]	49	9	-	-
HRWRA ** [kg/m^3^]	3.92	4.35	-	-

* GGBFS: ground granulated blast-furnace slag.

** HRWRA: high-range water-reducing admixture.

**Table 2:** Oxide composition of used materials (%).

Oxide composition	CaO	SiO_2_	Al_2_O_3_	Fe_2_O_3_	SO_3_	MgO	K_2_O	Na_2_O
Cement	65.47	17.71	4.50	3.37	3.44	3.29	1.11	0.16
Fly ash	11.70	56.70	17.70	5.90	1.80	1.80	1.20	1.10
GGBFS	44.30	33.70	11.60	1.20	1.50	4.30	0.40	0.20
